# Expression of endoglin (CD105) in cervical cancer

**DOI:** 10.1038/sj.bjc.6605009

**Published:** 2009-04-07

**Authors:** H J Zijlmans, G J Fleuren, S Hazelbag, C F Sier, E J Dreef, G G Kenter, A Gorter

**Affiliations:** 1 Department of Pathology, Leiden University Medical Centre, 2300 RC Leiden, The Netherlands; 2 Department of Obstetrics and Gynaecology, Medical Centre Haaglanden, 2501 CK The Hague, The Netherlands; 3 Department of Gastroenterology and Hepatology, Leiden University Medical Centre, 2300 RC Leiden, The Netherlands; 4 Department of Gynaecology, Leiden University Medical Centre, 2300 RC Leiden, The Netherlands

**Keywords:** cervical cancer, angiogenesis, cytokines

## Abstract

In this study, we have investigated the role of endoglin (CD105), a regulator of transforming growth factor (TGF)-*β*_1_ signalling on endothelial cells, basic fibroblast growth factor (bFGF) and vascular endothelial growth factor-A (VEGF-A) in cervical cancer. We have measured the number and determined the location of both newly formed (CD105-positive) and the overall number of (CD31-positive) blood vessels, and bFGF and VEGF-A expression using immunohistochemistry in 30 cervical carcinoma specimens. Vascular endothelial growth factor-A mRNA expression was determined using RNA-*in situ* hybridisation. CD105- and CD31-positive vessels and bFGF- and VEGF-A-positive cells were predominantly present in the stroma. The presence of CD105- and CD31-positive vessels in the stroma did neither correlate with the number of VEGF-A-positive cells nor the number of bFGF-positive cells. However, the number of CD105- and CD31-positive vessels was associated with the expression of *VEGF-A* mRNA in the epithelial cell clusters (*P*=0.013 and *P*=0.005, respectively). The presence of CD105-positive and CD31-positive vessels was associated with the expression of *α*v*β*6 (a TGF-*β*_1_ activator; *P*=0.013 and *P*=0.006, respectively). Clinically, the number of CD105-positive vessels associated with the number of lymph node metastasis (*P*<0.001). Furthermore, the presence of CD105-positive vessels within the epithelial cell clusters associated with poor disease-free survival (*P*=0.007).

Cervical cancer is the second most common cancer among women worldwide and one of the main causes of cancer-related death in the developing countries (Parkin *et al*, 2005). The prognosis of patients is, amongst others, dependent on tumour size, lymph node metastasis, capillary lymphatic space involvement, infiltration of the tumour in the cervix, infiltration of the tumour in the parametria and absence of tumour at the surgical margins (Hellebrekers *et al*, 1999). Cervical tumours are composed of malignant epithelial cells, tumour stroma, comprising the tumour vasculature, and an inflammatory infiltrate. A significant association between tumour vascular density and poor clinical outcome has been reported (Cooper *et al*, 1998, 1999; Pilch *et al*, 2001a). Growth and metastatic capacity of solid tumours depend on tumour vascular density and angiogenesis (Pilch *et al*, 2001b; Bremnes *et al*, 2006). Hypoxia in the centre of the epithelial cancer cell nests is thought to be a driving force behind angiogenesis (Byrne *et al*, 2005).

Tumours promote angiogenesis by secreting factors, like vascular endothelial growth factor-A (VEGF-A), basic fibroblast growth factor (bFGF) (Salgado *et al*, 2004; Bremnes *et al*, 2006) and transforming growth factor (TGF)-*β*_1_. In addition, the presence of an inflammatory infiltrate is thought to be of great importance in neovascularisation (Condeelis and Pollard, 2006).

A marker for angiogenesis is endoglin (Dallas *et al*, 2008). Endoglin, designated CD105, is a 90 kDa (under reducing conditions) TGF-*β* type III auxiliary receptor involved in the regulation of TGF-*β*_1_ signalling in endothelial cells. Overexpression of CD105 inhibits TGF-*β*/ALK5 signalling and TGF-*β*-induced growth inhibition, whereas knockdown of CD105 inhibits TGF-*β*/ALK1 signalling and endothelial cell proliferation (ten Dijke *et al*, 2008). CD105 is required for efficient TGF-*β*/ALK1 signalling on proliferating endothelial cells (ten Dijke and Arthur, 2007). In contrast to CD31, which is expressed on blood vessels both in normal tissue and in malignant lesions, endoglin is found predominantly on peritumoural and intratumoural blood vessels. The expression of CD105 on tumour-associated blood vessels makes CD105 a potential molecular target for therapy (Dallas *et al*, 2008).

Transforming growth factor-*β*_1_ plays a dual role in cancer: early in tumour development it acts as a tumour suppressor, inhibiting epithelial cell proliferation, whereas late in cancer development it suppresses the activity of the immune system and induces regulatory T cells (Elliott and Blobe, 2005). Transforming growth factor-*β*_1_ also plays an important role in angiogenesis by promoting proliferation and migration of endothelial cells at low TGF-*β*_1_ concentrations, whereas high concentrations lead to cytostasis and promote vessel maturation (ten Dijke and Arthur, 2007). Furthermore, TGF-*β*_1_ is known to induce VEGF expression (McMahon *et al*, 2006). Earlier we have reported on the expression of TGF-*β*_1_, plasminogen activator inhibitor (PAI)-1 and fibronectin (both target gene products of TGF-*β*_1_) and *α*v*β*6 (activator of TGF-*β*_1_) in cervical carcinoma (Hazelbag *et al*, 2002, 2004, 2007).

In this study, we have expanded our observations on the role of TGF-*β*_1_ in cervical cancer by measuring the number and determining the location of CD105-positive blood vessels. To further analyse the role of endoglin as a TGF-*β*_1_-associated regulatory molecule in the angiogenic process, we have compared the number and location of the newly formed CD105-positive vessels with the number and location of CD31-positive blood vessels (total number of blood vessels) and we have measured the expression of the pro-angiogenic factors, VEGF-A and bFGF. Finally, the clinico-pathological relevance of CD105 in cervical carcinoma was assessed.

## Materials and methods

### Patient material

A total of 30 patients treated with radical abdominal hysterectomy and bilateral pelvic lymph node dissection for uterine cervical cancer (Wertheim's procedure) was included in this study. According to the International Federation of Gynaecology and Obstetrics (FIGO) staging system for cervical carcinoma (Moore, 2006) 10 patients were selected for a high FIGO stage (at least FIGO IIA), whereas the other 20 patients were randomly selected patients with a FIGO stage IB. Patients had received no therapy before surgery. Treatment occurred between 1985 and 1994. Tissues had been fixed routinely in 10% v/v formalin and embedded in paraffin. Samples were used according to the guidelines of the Ethical Committee of the Leiden University Medical Centre. The patient characteristics are shown in Table 1.

### Immunohistochemistry

Immunohistochemical analysis was performed on 3 *μ*m paraffin sections, mounted on aminopropylethoxysilane-coated slides. Sections were deparaffinised, rehydrated and treated with 0.3% v/v H_2_O_2_ in methanol for 20 min to block endogenous peroxidase activity. Antigen retrieval was performed, if necessary, and sections were rinsed in phosphate-buffered saline (PBS) followed by an incubation with 1% w/v bovine serum albumin (BSA) in PBS. Subsequently, sections were stained for CD68 (Zijlmans *et al*, 2006), *α*ν*β*6 (Hazelbag *et al*, 2007), fibronectin (Hazelbag *et al*, 2002), VEGF-A, bFGF, matrix metalloproteinase (MMP)-2 (Sier *et al*, 2006), CD31 and CD105 (see Table 2 for characteristics of the primary antibodies). All antibodies were diluted in 1% BSA in PBS. In case of CD68, *α*ν*β*6, bFGF, MMP-2 and CD31 (a biotinylated secondary rabbit anti-mouse antibody) was used (1 : 200, DAKO, Glostrup, Denmark). For VEGF-A, biotinylated swine anti-rabbit antibody, and for fibronectin, biotinylated rabbit anti-goat antibody was used (both 1 : 400, DAKO). All slides were subsequently incubated with a biotinylated horseradish peroxidase (HRP)–streptavidin complex (1 : 100, DAKO) and immune complexes were visualised with diaminobenzidine. Staining for CD105 was performed with a CSA Detection System (DAKO), according to the manufacturer's protocol. Negative controls consisting of tissue sections where the primary antibody was replaced by an (irrelevant) antibody (DAKO) directed against the same isotype as the primary antibody.

### Probe preparation and RNA-*in situ* hybridisation

Oligonucleotide primers were chosen on the basis of known sequences (5′-GCCTCCGAAACCATGAACTTT-3′ (sense) and 5′-CCGCATAATCTGCATGGTGAT-3′ (antisense)) (GenBank accession number GI065522.1).

Cervical carcinoma sections were stained for V*EGF-A* mRNA as described earlier (Zijlmans *et al*, 2006). In short: 3 *μ*m paraffin sections were pre-treated and hybridised with 100 ng ml^−1^ DIG-labelled RNA probe diluted in hybridisation mixture containing 0.3 M NaCl and 0.03 M saline-sodium citrate (SSC). Hybridisation was allowed for 16 h at 50°C in a humidified chamber. Slides were washed for 30 min in 50% v/v formamide/2 × SSC at 42°C, followed by 45 min in 0.1 × SSC with 20 mM *β*-mercaptoethanol at 50°C and 30 min with 2 U ml^−1^ ribonuclease (RNase) T1 (Roche Diagnostics GmbH, Mannheim, Germany) in 2 × SSC, 1 mM EDTA at 37°C. RNA hybrids were detected using subsequently mouse anti-digoxigenin (1 : 2000, Sigma-Aldrich Chemie GmbH, Steinham, Germany), rabbit anti-mouse Ig (1 : 50, DAKO) and mouse alkaline phosphatase anti-alkaline phosphatase (APAAP, DAKO). The sense probe of each antisense V*EGF-A* mRNA probe served as a negative control. A cervical cancer sample stained for *TGF-β1* mRNA served as a positive control.

### Evaluation of immunohistochemical staining and RNA-*in situ* hybridisation

CD68-, VEGF- and bFGF-positive cells were quantitated by counting the number of stained cells per five, randomly selected, high-power fields (HPF, × 400). CD68-positive cells at the border of the epithelial–stromal interface were counted as present or absent. CD31- and CD105-positive vessels were quantitated by first searching for a high density of vessels at low magnification ( × 200, the so called ‘hot-spots’) in the stroma, followed by counting the number of positive vessels in five of these hot-spots at high magnification (HPF, × 400) (Weidner *et al*, 1991). The presence of CD105-positive vessels within the epithelial cell clusters or at the epithelial–stroma interface, were counted as present or absent. Fibronectin was scored at the epithelial–stroma interface as described by Havenith *et al* (1988), dividing the immunoreactivity in either <75% immunoreactivity or >75% immunoreactivity.

The staining of *VEGF-A* mRNA as well as VEGF-A protein, PAI-1, MMP-2 and *α*ν*β*6 in the tumour cells was scored as described earlier (Ruiter *et al*, 1998). Intensity was scored as none (0), weak (1), moderate (2) or strong (3) at low magnification ( × 100). Furthermore, the percentage of positive tumour cells was determined and divided into groups, numbered from 0 to 5: 0% (0, absent), 1–5% (1, sporadic), 6–25% (2, local), 26–50% (3, occasional), 51–75% (4, majority) and 76–100% (5, large majority). The two parameters were combined, representing the sum of both the percentage and the staining intensity of the positive cells, resulting in an overall score (0 or 2–8). Owing to low expression of *VEGF-A*, the scores were combined into two groups: category 0 (score 0, no expression), category 1 (score 2–8, expression present). Expression was scored by two independent researchers without knowing the identity and clinical outcome of patients.

### Statistical analysis

Data from immunohistochemistry as well as RNA-*in situ* hybridisation are given as the mean±s.d. Statistical analysis was performed using SPSS 14.0 (SPSS Inc., Chicago, IL, USA). Data were processed by using a *χ*^2^-test, the Mann–Whitney *U*-test or the Fisher's exact test, depending on number and distribution of the compared groups. Kaplan–Meier survival curves were generated to assess differences in disease-free survival (defined as the observation time in months from surgery to relapse of the disease) or cumulative overall survival (defined as time in months from surgery to death owing to cervical cancer). *P*<0.05 was considered statistically significant.

## Results

### Number and location of CD105- and CD31-positive blood vessels

**Figure 1:**
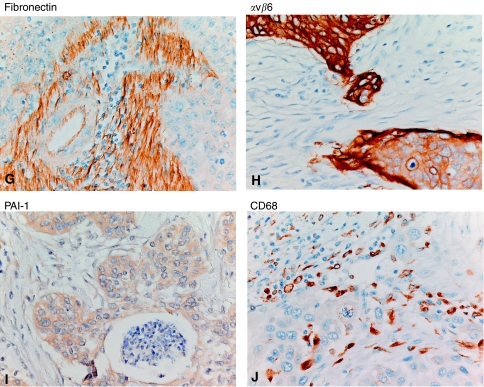
Expression and location of CD105- and CD31-positive vessels, bFGF, VEGF fibronectin, *α*v*β*6, PAI-1 and CD68-positive cells. Expression and location of CD105- and CD31-positive vessels, bFGF and VEGF-A were determined using immunohistochemistry as well as RNA-*in situ* hybridisation as described in Materials and Methods ( × 125 magnification). (**A**) CD105 vascular staining, A1. Detail ( × 400 magnification) of vessels present in the tumour stroma as well as in the epithelial cell clusters, (**B**) CD31vascular staining, B1. Detail ( × 400 magnification) of vessels present in the tumour stroma as well as in the epithelial cell clusters. (**C**) bFGF, positive staining of the border of the epithelial cell clusters and cells in the stromal compartment; (**D**) VEGF-A immunohistochemical staining with increased positive staining of the borders of the epithelial cell clusters, D1. Detail ( × 400 magnification) of VEGF-A-positive stromal cells, (**E**) *VEGF-A* RNA-*in situ* hybridisation with weak cytoplasmic staining of the epithelial cell clusters, (**F**) Negative (sense) control of *VEGF-A* RNA-*in situ* hybridisation. Expression and location of fibronectin, *α*v*β*6, PAI-1 and CD68-positive cells ( × 400 magnification). (**G**) fibronectin, positive staining (>75%) of the stromal compartment. (**H**) *α*v*β*6, positive staining (strong intensity; of the border) of the epithelial cell clusters. (**I**) PAI-1, positive staining of the epithelial cell clusters. (**J**) Positive staining of CD68-positive cells in the stromal compartment and in the epithelial cell clusters.

**Figure 2:**
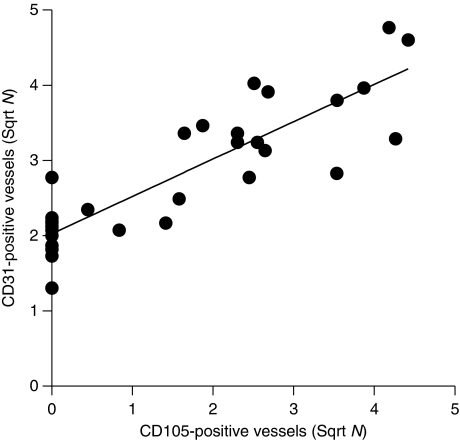
Correlation between CD105- and CD31-positive vessels. Number of stromal CD105- and CD31-positive vessels was determined using immunohistochemistry as described in Materials and Methods (Pearson, *r*^2^=0.747, *P*<0.001).

First, we have investigated the relationship between the number and location of (newly formed) blood vessels, using anti-CD105 and anti-CD31 (providing an estimate of the total number of blood vessels) monoclonal antibodies (Figure 1A and B, respectively). The number of newly formed stromal CD105-positive vessels (mean 5±1) was lower than the number of stromal CD31-positive vessels (mean 9±1). The number of CD105-positive vessels ranged from 0 to 20, whereas the number of CD31-positive vessels ranged from 2 to 23. In 8 out of 30 cases CD105-positive vessels were located within the epithelial cell clusters, whereas in 18 out 30 cases CD31-positive vessels were observed within these clusters. There was a significant correlation between the expression of CD105- and CD31-positive vessels in the stroma (*r*^2^=0.747, *P*<0.001; Figure 2).

### Number and location of VEGF-A and bFGF-positive cells

**Figure 3:**
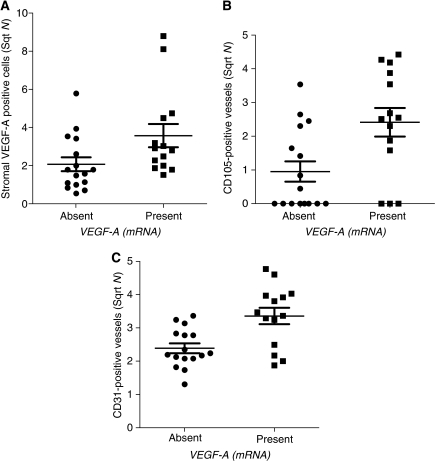
Association between *VEGF-A* (mRNA) expression measured in the epithelial cell clusters and the number of VEGF-A-positive cells and blood vessels. (**A**) Correlation between *VEGF-A* (mRNA) expression measured in the epithelial cell clusters and the number of VEGF-A-positive cells (Mann–Whitney *U*-test, *P*=0.048) in tumour stromal compartment. (**B**) Correlation between *VEGF-A* (mRNA) expression measured in the epithelial cell clusters and the number of stromal CD105-positive vessels (Mann–Whitney *U*-test, *P*=0.013). (**C**) Correlation between *VEGF-A* (mRNA) expression measured in the epithelial cell clusters and the number of stromal CD31-positive vessels (Mann–Whitney *U*-test, *P*=0.005).

Subsequently, we have investigated the number and location of bFGF- and VEGF-A-positive cells (Figure 1C and D, respectively). Basic fibroblast growth factor-positive cells were predominantly observed in the stroma. The number of bFGF-positive cells ranged from 0 to 71 (mean 24±5). Only 3 out of 30 cases showed bFGF expression within the epithelial cell nests. These cells were mainly located at the epithelial–stromal interface. Another six tumours showed bFGF-positive cells within the necrotic centres of epithelial nests, whereas tumour cells showed a negative staining. Vascular endothelial growth factor-A was both measured at the protein level using with immunohistochemistry and at the mRNA level using RNA-*in situ* hybridisation (Figure 1E). Vascular endothelial growth factor-A-positive cells were mainly observed in the stroma. The number of VEGF-A-positive cells ranged from 0 to 77 (mean 11±3). Weak VEGF-A protein expression was observed throughout the epithelial cell clusters with an increased intensity at the epithelial–stromal interface (16 out of 30 cases; Figure 1D). In addition, *VEGF-A* (mRNA) expression was observed in the epithelial cell clusters (14 out of 30 cases). Expression of *VEGF-A* (mRNA) within the epithelial cell clusters did not correlate with VEGF-A protein expression within the epithelial cell clusters, but did associate with the number of VEGF-A-positive cells in the stroma (*P*=0.048, Figure 3A).

### Association between blood vessels and bFGF and VEGF-A

No significant association between the presence of CD105-positive vessels in the tumour or the total number of CD105-positive and CD31-positive vessels in the stroma, and the number of bFGF- or VEGF-A-positive cells in the stroma was observed (data not shown). However, both the number of CD105-positive and the number of CD31-positive vessels in the stroma associated significantly with expression of *VEGF-A* (mRNA) within the epithelial cell clusters (*P*=0.013 (Figure 3B) and *P*=0.005 (Figure 3C), respectively).

### Association between blood vessels and tumour-associated macrophages

**Figure 4:**
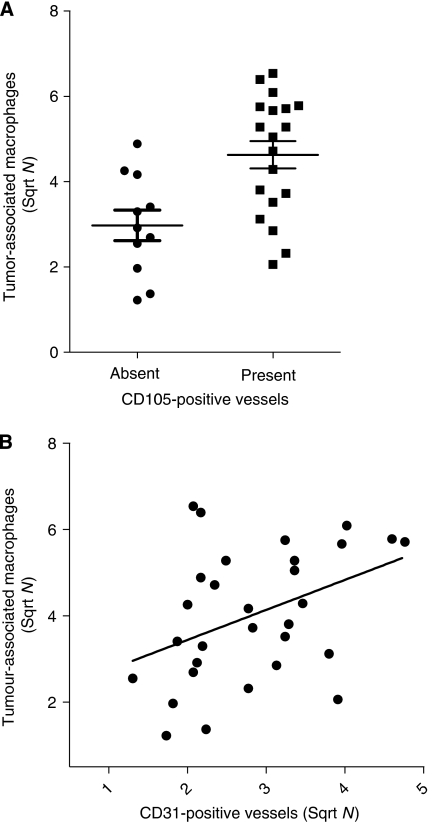
Association between tumour-associated macrophages and blood vessels. (**A**) Association between the number of total (stroma and epithelial cell clusters) tumour-associated macrophages and the presence of CD105-positive vessels (Mann–Whitney *U*-test, *P*=0.004). (**B**) Correlation between the total number of tumour-associated macrophages and the number of stromal CD31-positive vessels (Pearson, *r*^2^=0.150, *P*=0.034).

As angiogenic factors are also produced by tumour-associated macrophage (TAM), we have enumerated the number of CD68-positive cells (Zijlmans *et al*, 2007) and correlated their number with the number of CD105- and CD31-positive stromal vessels. The presence of CD105-positive vessels in the tumour was significantly associated with total (stroma and epithelial cell clusters) number of CD68-positive cells (*P*=0.004; Figure 4A). However, the number of CD105-positive vessels in the stroma was not significantly correlated with the total number of CD68-positive cells. In addition, there was a significant correlation between the number of CD31-positive vessels in the stroma and the total number of CD68-positive cells (*r*^2^=0.150, *P*=0.034; Figure 4B).

### Association between blood vessels and TGF-*β*_1_ target gene products PAI-1 and fibronectin and the TGF-*β*_1_ activator *α*ν*β*6

**Figure 5:**
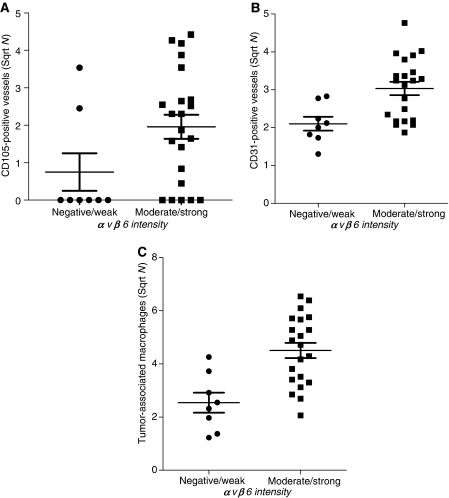
Association between *α*v*β*6 expression and CD31-positive vessels and tumour-associated macrophages. (**A**) Association between the presence of moderate/strong *α*v*β*6 intensity and the number of stromal CD105-positive vessels (Mann–Whitney *U*-test, *P=*0.053). (**B**) Association between the presence of moderate/strong *α*v*β*6 intensity and the number of stromal CD31-positive vessels (Mann–Whitney *U*-test, *P=*0.006). (**C**) Association between the presence of moderate/strong *α*v*β*6 intensity and the total number of tumour-associated macrophages (Mann–Whitney *U*-test, *P=*0.002).

To explore the role of TGF-*β* in the generation of CD105-positive vessels both the presence of CD105-positive blood vessels in the epithelial cell clusters and the number of blood vessels in the stroma were associated with the presence of PAI-1 in the epithelial cell clusters (Hazelbag *et al*, 2004) and the amount of fibronectin in the stroma (Hazelbag *et al*, 2002), both target genes of TGF-*β*_1_. Neither the presence of CD105-positive vessels within the epithelial cell clusters nor the number of stromal CD105-positive vessels associated significantly with PAI-1 expression (data not shown). However, an association between the number of stromal CD31-positive vessels and high levels of fibronectin (>75%) was found (*P*=0.038; data not shown). Furthermore, a significant association between the number of stromal CD31-positive vessels and moderate or strong staining intensity (*vs* no or weak staining intensity at the epithelial–stromal interface) of *α*ν*β*6 was observed (*P*=0.006; Figure 5B). Although a difference between the number of stromal CD105-positive vessels and moderate or strong staining intensity (*vs* no or weak staining intensity at the epithelial–stromal interface) of *α*ν*β*6 was observed (Figure 5A), this difference was not significant (*P*=0.053). In addition, an association between the total number of TAM and *α*ν*β*6 staining intensity was found (*P*=0.002; Figure 5C).

### Association between blood vessels and clinico-pathological parameters

Finally, we have associated both the presence of CD105-positive blood vessels within the epithelial cell clusters and the number of stromal CD105- or CD31-positive blood vessels with the FIGO stage, lymph node metastasis, tumour size, infiltration depth, vascular space involvement, parametrial invasion, human papillomavirus (HPV) status and histology. No significant associations were found between the majority of these parameters and the presence of CD105-positive blood vessels within the epithelial cell clusters or the number of stromal blood vessels. Only the presence of CD31-positive vessels in the epithelial cell clusters associated significantly with vascular space involvement (Fisher's exact test, *P*=0.021).

The number of both CD105- and CD31-positive stromal vessels was significantly associated with the presence of positive lymph nodes (*P*=0.005 and *P*=0.011, respectively).

**Figure 6:**
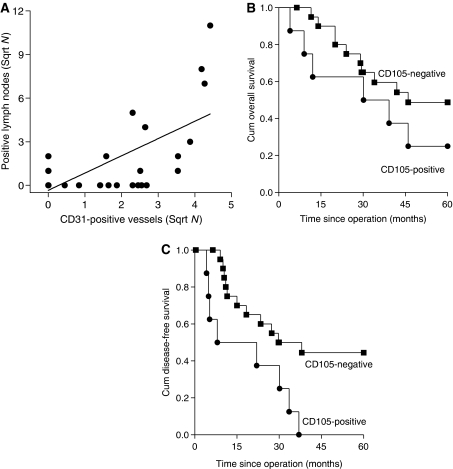
Association between CD105-positive vessels, the number of positive lymph nodes and survival. (**A**) Correlation between the number of stromal CD105 positive vessels and the number of positive lymph nodes (*r*^2^=0.615; *P*<0.001), (**B**) Overall survival (Kaplan–Meier, logrank 1.55, *P*=0.214), (**C**). Disease-free survival (Kaplan–Meier, logrank 7.28, *P*=0.007), both stratified by presence of CD105-positive vessels within the epithelial cell clusters of cervical carcinoma. • CD105 present in epithelial cell clusters, ▪ CD105 absent in epithelial cell clusters.

In addition, the number of CD105-positive stromal vessels was significantly associated with the number of positive lymph nodes (*P*<0.001; Figure 6A). Finally, the presence of CD105-positive vessels within the epithelial cell clusters showed a negative relationship with disease-free survival (Figure 6C; *P*=0.007). Sample size was too small to calculate whether or not this is an independent prognostic variable.

## Discussion

In this study, we have investigated the role of endoglin (CD105), a regulator of TGF-*β* signalling on endothelial cells, bFGF and VEGF-A expression in 30 cervical carcinoma specimens. Most vessels were detected in the stroma as could be expected, as a pre-existing vascular network is necessary for developing new vessels (Hicklin and Ellis, 2005). Only one recent study has reported on the expression of CD105 in cervical cancer (Mazibrada *et al*, 2008). In agreement with this study, we also observed a positive correlation between the number of (newly formed) CD105- and (total number) CD31-positive vessels. In addition, we observed both CD105- and CD31-positive vessels within the epithelial cancer cell clusters. As blood vessels originate from the vasculature in the stroma, we assume that these vessels are embedded in thin layer of stroma and surrounded by epithelial cancer cells.

Subsequently, we have assessed the association between the number of CD105-vessels and bFGF- and VEGF-A expressing cells. We did not observe an association between bFGF-expressing cells with the number of CD105 (or CD31)-positive vessels. An increase in *bFGF* mRNA expression has been reported during cervical tumour development (Fujimoto *et al*, 1997; Van Trappen *et al*, 2002), whereas a decrease in *bFGF* expression was reported in the study of Soufla *et al* (2005). To our knowledge, our study is the first study on bFGF-expressing cells in cervical carcinoma at the protein level using immunohistochemistry. Our study confirmed the absence of a statistical significant association between *bFGF* and *VEGF-A* expression in cervical cancer (data not shown) as earlier shown by Van Trappen *et al* (2002) using PCR-analysis.

A negative association between VEGF-A protein expression and microvessel density, was reported by Tjalma *et al* (2000) in a study comprising 152 cervical carcinoma patients. In our study, we did not find an association between either the number of CD105 (or CD31)-positive vessels (microvessel density) and VEGF-A protein expression. In contrast to the study of Tjalma *et al* (2000), in our study we have also determined the presence of *VEGF-A* mRNA expressing cells. We were able to show a positive association between expression of *VEGF-A* mRNA and both CD105 expression and CD31 expression, suggesting that expression of *VEGF-A* mRNA is positively associated with microvessel density. The associations remained significant even after a Bonferroni's correction for multiple testing by a factor 3. The presence of *VEGF-A* mRNA only detectable in the centre of the epithelial cell clusters, suggests that this is caused by hypoxia (Lee *et al*, 2008). Although VEGF-A is considered to be one of the most important factors involved in angiogenesis (Hicklin and Ellis, 2005), surprisingly VEGF-A protein present at the epithelial–stromal interface did not correlate with the number of CD105 (or CD31)-positive vessels. This might be explained by consumption of VEGF-A protein by target cells.

The role of TAM in pressing an angiogenic switch has been highlighted by Lin and Pollard (2007), suggesting that TAM significantly contribute to angiogenesis. Tumour-associated macrophages are known to contain many proangiogenic factors, such as VEGF (Leek *et al*, 2000), TNF-*α* (Pusztai *et al*, 1994), CXCL-8 (Fujimoto *et al*, 2000), bFGF (Stupack *et al*, 1999) and proteases such as MMP-2 and MMP-9 (Pollard, 2004). These MMPs are able to free pro-angiogenic cytokines, such as TGF-*β* and VEGF-A, from the matrix. Indeed, we could show a positive association between the presence of CD105-positive vessels in the tumour, the stromal number of CD31-positive vessels and the total number of TAM. In contrast, an earlier study by Davidson *et al* (1999), using CD31 as a vessel marker did not find this association. Our results suggest that that the presence of TAM is associated with neo-angiogenesis. However, there was no significant relationship between the number of VEGF-A-positive cells in stroma or VEGF-A protein in the epithelial cancer cell clusters and the number of TAM. This suggests that other factors in addition to VEGF-A contribute to angiogenesis. These factors may be induced by cytokines from TAM or liberated from the extracellular matrix by MMPs derived from TAM. In addition, we did not observe a significant association between MMP-2 expressed at the border between the epithelial cancer cells and VEGF-A protein (Sier *et al*, 2006).

Transforming growth factor-*β*_1_ plays an important role in angiogenesis, by promoting proliferation and migration of endothelial cells or promoting vessel maturation (ten Dijke and Arthur, 2007). Furthermore, TGF-*β*_1_ is known to induce VEGF expression (McMahon *et al*, 2006). We did not observe an association between the presence or the number of CD105-positive vessels and PAI-1, fibronectin (surrogate markers of TGF-*β*_1_ activity), *α*ν*β*6 and MMP-2 (activators of TGF-*β*_1_) expression. However, the total number of (CD31-positive) vessels correlated with the expression of *α*ν*β*6. In addition, *α*ν*β*6 correlated significantly with the amount of fibronectin (*P*=0.037, data not shown), which is one of the factors that supports blood vessel growth (Hazelbag *et al*, 2007). Interestingly, *α*ν*β*6 is also known to upregulate MMP-9 expression and thus may also contribute to the release of VEGF-A from the extracellular matrix (Scott *et al*, 2004).

**Figure 7:**
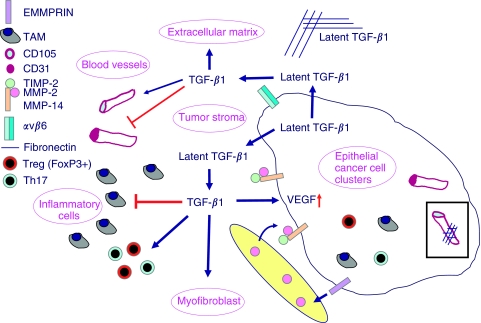
Role of TGF-*β*_1_ in cervical cancer. Cervical tumours are composed of malignant epithelial cells, tumour stroma, comprising the tumour vasculature extracellular matrix and (myo)fibroblasts, and an inflammatory infiltrate. Latent TGF-*β*_1_ is produced amongst others by cervical cancer cells, secreted and stored in the extracellular matrix. TGF-*β*_1_ can be activated by the epithelial cell specific integrin *ανβ*6 or by matrix metalloproteinases (MMPs), especially active MMP-2 in complex with MMP-14 and TIMP-2 on the cell membrane of cervical cancer cells at the epithelial cell–stroma border. Active TGF-*β*_1_ induces VEGF in the epithelial cancer cells and differentiates fibroblasts into myofibroblasts. Depending on the local concentration, active TGF-*β*_1_, promotes endothelial proliferation and migration and promotes cytostatis and vessel maturation. Active TGF-*β*_1_ acts as an immunosuppressor by blocking the activity of the inflammatory cells and inducing FoxP3-positive regulatory cells and Th17 cells.

On the basis of all our findings, we suggest that TGF-*β*_1_ also plays a central role in the progression of cervical carcinoma (Figure 7). Latent TGF-*β*_1_ is produced amongst others by cervical cancer cells, secreted and stored in the extracellular matrix. This TGF-*β*_1_ is then processed by either the epithelial cell specific integrin *α*ν*β*6 (Sheppard, 2005; Hazelbag *et al*, 2007) or by MMPs, especially active MMP-2 (Sier *et al*, 2006) in complex with MMP-14 and TIMP-2 on the cell membrane of cervical cancer cells at the epithelial cell–stroma border. Active TGF-*β*_1_ can induce VEGF in the epithelial cancer cells and differentiates fibroblasts surrounding the epithelial cancer cells into myofibroblasts. Furthermore, active TGF-*β*_1_ depending on the local concentration either promotes endothelial proliferation and migration or promotes cytostatis and vessel maturation. In addition, active TGF-*β*_1_ acts as an immunosuppressor by blocking the activity of the inflammatory cells and inducing FoxP3-positive regulatory cells (Jordanova *et al*, 2008) and Th17 cells (Manel *et al*, 2008).

The prognostic value of CD105 and CD31 has been assessed earlier in breast, colon and ovarian carcinomas (Dales *et al*, 2004; Erdem *et al*, 2006; Minhajat *et al*, 2006; Dhakal *et al*, 2008). High expression of CD105 correlated with increased risk of metastasis in lymph node-positive breast carcinoma patients (Dales *et al*, 2004). In this latter study, CD105 was shown to have an improved predictive value compared with CD31. In agreement with this study, in cervical carcinoma we observed an association between the number of CD105-positive vessels and the number of positive lymph nodes. With respect to survival, only the presence of newly formed vessels (CD105) within the epithelial cell clusters was correlated with a poor disease-free survival.

Our study suggests that the presence of CD105-positive vessels in the epithelial cell clusters may be of use as a (poor) prognostic factor in cervical carcinoma. This is important as CD105 has been proposed as a marker of tumour vasculature and a potential target for therapy of cervical carcinoma (Dallas *et al*, 2008).

## Figures and Tables

**Table 1:** Summary of clinico-pathological features of patients and tumours

**Characteristics of patients and tumours**	**Outcome**	***N*** a
Age	45 (mean)	30
	29–72 (range)	
FIGO stage	⩽IB	20
	⩾IIA	10
Lymph node metastasis	No	16
	Yes	14
Tumour size^b^	<40 mm	16
	⩾40 mm	12
Infiltration depth^b^	<15 mm	15
	⩾15 mm	9
Vascular space involvement	No	10
	Yes	20
Parametrial invasion	No	20
	Yes	10
HPV status^b^	16, 18	19
	Other	6
Histology	Squamous	25
	Adenosquamous	4
	Adeno	1

FIGO=Federation of Gynaecology and Obstetrics.

a *N*=number of patients/cervical carcinomas.

b Cases missing.

**Table 2:** Characteristics of used primary antibodies

**Antigen**	**Clone**	**Source**	**Directed against**	**Antigen retrieval**	**Dilution**	**Incubation conditions**	**Manufacturer**
*α*ν*β*6	2G2	Mouse	*β*6	Citrate 0.01 M	1 : 2000	on 4°C	Biogen Idec, Cambridge, MA, USA
bFGF	6	Mouse	bFGF	Citrate 0.01 M	1 : 600	on 4°C	BD Biosciences, Franklin Lakes, NJ, USA
CD31	JC/70A	Mouse	Endothelial cells	Citrate 0.01 M	1 : 400	on RT	Neomarkers, Fremont, CA, USA
CD68	KP-1	Mouse	Macrophages	Trypsin 0.1% w/v	1 : 1600	on^a^ RT^b^	Dako, Glostrup, Denmark
CD105	SN6H	Mouse	Endoglin	None	1 : 2000	1 h RT	Dako, Glostrup, Denmark
Fibronectin		Goat	Fibronectin	Pepsin 0.4% w/v	1 : 1000	on RT	Sigma, St Louis, MO, USA
MMP-2	CA-4001	Mouse	Proform of MMP-2	None	1 : 200	on RT	Neomarkers, Fremont, CA, USA
VEGF-A		Rabbit	VEGF-A	Pepsin 0.4% w/v	1 : 100	2 h RT	Santa Cruz Biotechnology Inc., Santa Cruz, CA, USA

bFGF=basic fibroblast growth factor; VEGF=vascular endothelial growth factor-A.

a On=overnight.

b RT=room temperature.
